# circRNA CDR1as Regulated the Proliferation of Human Periodontal Ligament Stem Cells under a Lipopolysaccharide-Induced Inflammatory Condition

**DOI:** 10.1155/2019/1625381

**Published:** 2019-09-08

**Authors:** Fang Wang, Xi Chen, Ying Han, Shuang Xi, Guofeng Wu

**Affiliations:** ^1^Key laboratory of Shaanxi Province for Craniofacial Precision Medicine Research & Department of Prosthodontics, College of Stomatology, Xi'an Jiaotong University, Xi'an, Shaanxi, China; ^2^Department of Prosthodontics, Digital Stomatological Engineering Center, Nanjing Stomatological Hospital, Medical School of Nanjing University, Nanjing, China; ^3^Department of Prosthodontics, Nanjing Stomatological Hospital, Medical School of Nanjing University, Nanjing, Jiangsu, China

## Abstract

circRNA CDR1as (CDR1as) has been demonstrated to play important roles in a variety of inflammation-related diseases by acting as miRNA sponges. The present study is aimed at investigating the potential roles of CDR1as in the proliferation of human periodontal ligament stem cells (PDLSCs) under an inflammatory condition induced by *Porphyromonas gingivalis*-derived lipopolysaccharide (LPS). Human periodontal ligament cells (PDLCs) were isolated from periodontal ligament tissue, and PDLSCs were sorted from PDLCs based on the STRO-1 expression through fluorescence-activated cell sorting. We further found that CDR1as was significantly downregulated in LPS-treated PDLSCs compared to untreated cells, as well as in normal periodontal ligament tissues compared to periodontitis tissues. Knockdown of CDR1as promoted LPS-induced proliferative inhibition of PDLSCs, whereas overexpression of CDR1as alleviated the LPS-induced proliferative ability of PDLSCs. Mechanistically, CDR1as functioned as an miR-7 sponge to activate the ERK signal pathway to mediate the inhibition effect of LPS on cell proliferation. Taken together, our findings revealed the effects of the interacting pair of CDR1as/miR-7 on the proliferation ability of PDLSCs within their surrounding inflammatory microenvironment of periodontitis.

## 1. Introduction

Periodontitis is the most prevalent chronic inflammatory disease in the tooth-supporting tissues caused by the accumulation of subgingival plaque and the action of specific periodontopathogenic bacteria [1]. *Porphyromonas gingivalis* (*P. gingivalis*) is one of the main bacteria associated with chronic periodontitis [2]. Chronic periodontitis leads to the destruction of periodontal ligament, cementum, and bone [3]. Periodontal ligament stem cells (PDLSCs) are a unique population of mesenchymal stem cells (MSCs) and have been successfully isolated from human periodontal ligament by researchers through different methods including traditional plastic adherence and immunomagnetic selection based on the expression of MSC-associated surface markers STRO-1 antigen, CD146, CD29, CD44, and CD106 [4]. PDLSCs exhibit osteogenic potentials both under inductive culture conditions in vitro and PDLSCs transplantation therapies in animal models [5]. PDLSCs have been showed to produce typical periodontal ligament-like tissue to regenerate tissue damaged by periodontitis [6], which provides a new prospect for periodontal tissue regeneration. Periodontal tissue regeneration has close relationships with abilities of cell proliferation, migration, and osteo/dentinogenic differentiation of MSCs in an inflammatory niche [7]. In performing their periodontal tissue regenerative activity when inflammation is regarded as the reason for tissue damage, PDLSCs interact with their surrounding inflammatory microenvironment [4]. Thus, rescuing the impaired function of MSCs, such as proliferation ability, in periodontitis is the key for treatment, especially in a manner independent of exogenous MSCs.

Circular RNAs (circRNAs) comprise a novel class of noncoding RNAs that play important roles in a variety of diseases by mediating alternative splicing or acting as miRNA sponges, such as osteoarthritis [8, 9] and atherosclerosis [10]. Emerging evidence offered a putative role of some circRNAs in osteogenic differentiation [11]. Moreover, several circRNAs were predicted and identified to have potential roles in the osteogenesis of PDLSCs [6]. CDR1as, also known as CiRS-7, a circRNA sponge for miR-7, was confirmed to have around 70 conserved seed matches for miR-7 and to act as an miR-7 sponge to perform biological functions [12, 13]. Accumulating reports suggested that CDR1as is involved in various physiological and pathological processes through a variety of pathways. Functional investigations have showed that the CDR1as-miR-7 axis can regulate the development of nervous systems [13], insulin transcription and secretion in islet cells [14], and the process of various types of cancer, including colorectal cancer, hepatocellular carcinoma, and melanoma [15]. Moreover, circular RNA CDR1as regulates osteoblastic differentiation of PDLSCs via the miR-7/GDF5/SMAD and p38 MAPK signaling pathways [16]. Further functional studies proved that CDR1as exerts its promoted or inhibited effects on cell proliferation among different types of carcinoma [17–19]. Therefore, it is interesting to investigate whether CDR1as has potential roles to regulate the proliferation of PDLSCs to regenerate tissue and teeth damaged by periodontitis.

In this study, we investigated the role of CDR1as in the proliferation of PDLSCs, as well as the mechanisms involved, under an inflammatory condition induced by *P gingivali*s-derived LPS, the major component of the outer membrane of gram-negative bacteria and a pertinent deleterious factor in the oral microenvironment.

## 2. Materials and Methods

### 2.1. Human Periodontal Ligament Tissue Collection and Primary Cell Culture

Human periodontal ligament tissues were obtained from healthy teeth for orthodontic purpose or from diseased teeth with chronic periodontitis after tooth extraction. The study was approved by the local ethics committee of the Xi'an Jiaotong University (Xi'an, Shaanxi, China), and the consent was obtained from donors. All donors were aged between 30 and 40 years and free of systemic disease. They had not received antibiotic therapy in the last 3 months. The clinical diagnosis of donors was based on visual and radiographic assessment of the periodontal tissues. Tissues were gently scraped from the surfaces of the root, washed and cut into small pieces with scissors, and then minced completely using sterile scalpel blades, followed by incubation with type I collagenase at 0.75 mg/ml for 30 min to isolate human periodontal ligament cells (PDLCs) as described previously [3]. Single-cell suspensions obtained after enzymic digestion and a strainer filtration were seeded in 10 cm petri dishes containing *α*-MEM supplemented with 15% FBS, 2 mmol/L-glutamine, 100 U/ml penicillin, and 100 mg/ml streptomycin and incubated at 37°C in 5% CO_2_.

### 2.2. Flow Cytometry and Fluorescence-Activated Cell Sorting (FACS)

Primary PDLCs were firstly examined expressions of hematopoietic stem cell (HSC) markers CD34 and CD45 by flow cytometry. Briefly, at passage 3, PDLCs were routinely harvested and blocked with FcR blocking reagent (Miltenyi Biotec, Auburn, CA) at 4°C for 10 min. Then, PDLCs were incubated with anti-human CD34-phycoerythrin (PE) or CD45-PE (BD Pharmingen, San Diego, CA) for 20 min on ice in the dark. After washing twice, cells were resuspended in HBSS/2% FBS (Sigma). The suspension was filtered through a 70 mm nylon mesh. Samples were analyzed on a FACSCalibur flow cytometer (Becton Dickinson, San Jose, CA).

To further isolate stem cells from PDLCs, single-cell suspension of primary cells was sorted through immunomagnetic selection of FACS based on the expression of MSC-associated surface marker STRO-1 antigen. Harvested cells were incubated with human MSC positive maker of STRO-1-PE (BD Pharmingen) for 20 min on ice in the dark, followed by washing and resuspension in HBSS/2% FBS containing 1 mg/ml 7-AAD. Finally, samples were analyzed and sorted in a BD FACSCalibur flow cytometer (BD Biosciences, NJ). After sorting, STRO-1+ cells were considered as PDLSCs and maintained in complete medium, consisting of *α*-MEM supplemented with 15% FBS, 2 mmol/L-glutamine, 100 U/ml penicillin, and 100 mg/ml streptomycin, at 37°C in 5% CO_2_.

### 2.3. RNA Extraction and Real-Time Quantitative-PCR (RT-qPCR)

Total RNA from tissues or cell lines was extracted using TRIzol reagent (TransGene, Shanghai, China), and RT-qPCR was performed according to the manufacturer's instructions of iQ5 light cycler (Bio-Rad, Hercules, CA). The 2^−*ΔΔ*Ct^ method was used to estimate relative expression changes of genes of CDR1as and miR-7. The expression levels of CDR1as and miR-7 were normalized to *β*-actin and U6, respectively. The sequences of primers for RT-qPCR were as follows: CDR1as, 5′-TCTGCTCGTCTTCCAACATC-3′ (forward) and 5′-AGATCAGCACACTGGAGACG-3′ (reverse); GAPDH, 5′-CGACAGCAGCCGCATCTT-3′ (forward) and 5′-CCAATACGACCAAATCCGTTG-3′ (reverse); miR-7, 5′-TGGAAGACTAGTGATTTTGTTG-3′ (forward) and 5′-ACGCTGGAAGACTAGTGATTTTG-3′ (reverse); and U6, 5′-CTCGCTTCGGCAGCACA-3′ (forward) and 5′-AACGCTTCACGAATTTGCGT-3′ (reverse).

### 2.4. Inflammatory Induction of PDLSCs

For inflammatory induction, 80% confluent PDLSCs were cultured in complete medium containing *P. gingivalis*-derived LPS (Sigma-Aldrich China Inc., Shanghai) at final concentrations of 10 *μ*g/ml for 3 h, 6 h, and 12 h, according to recommended concentration, and tumor necrosis factor-alpha (TNF-*α*) released in medium in the treated cells was used as indicator for LPS-induced immune response.

The secretion of TNF-*α* in the medium was measured using ELISA. PDLSCs were cultured in complete medium for another 24 h after treatment with LPS. Then, the medium was collected, and cell debris was removed via centrifugation. The level of TNF-*α* secreted in the medium was measured using an ELISA kit of TNF-*α* (Huzhen Biological Technology Co. Ltd., Shanghai, China) in accordance with the manufacturer's instructions.

### 2.5. Cell Proliferation Assay

Cell proliferation was examined using the Cell Counting Kit-8 (CCK-8, Beyotime, Shanghai, China) in accordance with the manufacturer's protocol. Briefly, cells were plated in 96-well plates at the same density of 2 × 10^3^ cells/well and cultured for 0, 1, 2, and 3 days. At the indicated time point, CCK-8 solution at a medium dilution of 1 : 10 diluted was added to each well and the plate was incubated at 37°C for 3 hours. The absorbance was measured by a microplate reader (Bio-Rad, Hercules, CA) at a wavelength of 450 nm. Cell numbers were calculated in reference to a standard curve obtained under the same conditions.

### 2.6. Transient Transfection

Transfection was conducted when PDLSCs reached 70-80% confluence using Lipofectamine 3000 (Invitrogen, Carlsbad, CA) according to the manufacturer's protocol. The miR-7 mimic, miR-7 inhibitor, si-CDR1as, pcdna3.1-circ-mini-CDR1as, and corresponding negative controls were transfected separately or cotransfected. The cells were collected 48 h or 72 h after transfection for mRNA or protein detection, respectively. All reagents were purchased from GenePharma (Shanghai, China). The sequences of these RNA oligoribonucleotides were as follows: miR-7 mimic, 5′-UGGAAGACUAGUGAUUUUGUUGU-3′ (forward) and 5′-AACAAAAUCACUAGUCUUCCAUU-3′ (reverse); miR-7 inhibitor, 5′-ACAACAAAAUCACUAGUCUUCCA-3′; si-CDR1as, 5′-GGUCUUCUAAUAUCUCCAATT-3′ (forward) and 5′-UUGGAGAUAUUAGAAGACCTT-3′ (reverse); miR-NC, 5′-CAGUACUUUUGUGUAGUACAA-3′; and si-NC, 5′-UUCUCCGAACGUGUCACGUTT-3′ (forward) and 5′-ACGUGACACGUUCGGAGAATT-3′ (reverse). The expression plasmid for expressing CDR1as sequence was DNA3.1(+) CircRNA Mini Vector, a gift from Jeremy Wilusz (Addgene plasmid # 60648).

### 2.7. Western Blotting

Total proteins were extracted from PDLSCs using PRO-PREP Protein Extraction Solution (iNtRON Biotechnology Inc., Gyeonggi-do, Korea) according to the manufacturer's instructions. The protein content was determined with the Bradford Easy Protein Quantitative Kit (TransGene). Equal amounts of protein extracts in lysis buffer were subjected to SDS-PAGE on 4-12% polyacrylamide gels then transferred to PVDF membranes. Membranes were incubated with primary antibodies against total-ERK, phospho-ERK, and GAPDH at 4°C overnight. After being washed with TBST, the membranes were incubated with HRP-conjugated secondary antibodies for 2 h at room temperature. Finally, immunoreactive proteins were visualized with an ECL detection kit (Beyotime).

### 2.8. Statistical Analysis

Results were reported as mean ± SD. All data were obtained from at least three independent experiments. All statistical analyses were performed using ANOVA (SPSS 11.5, IBM Corporation, Armonk, NY). Statistically significant difference was considered at *p* < 0.05.

## 3. Results

### 3.1. Isolation and Identification of PDLSCs

**Figure 1:**
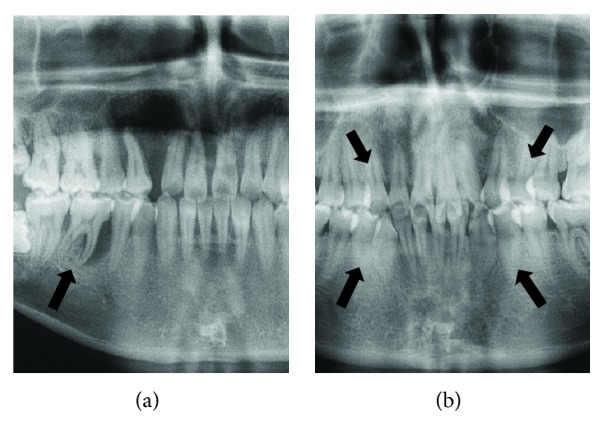
Representative intraoral dental radiographs of donors. (a) Radiograph of a donor with periodontitis shows severe periodontal loss. (b) Radiograph of a healthy donor undergoing teeth extractions for orthodontic treatment purposes. The arrows indicate the teeth that will be extracted.

Periodontal ligament tissues were obtained from donors with or without periodontitis, and the clinical diagnosis was confirmed by visual and radiographic assessment of periodontal tissues of donors. Representative radiograph from donors with or without periodontitis can be seen in Figure 1.

**Figure 2:**
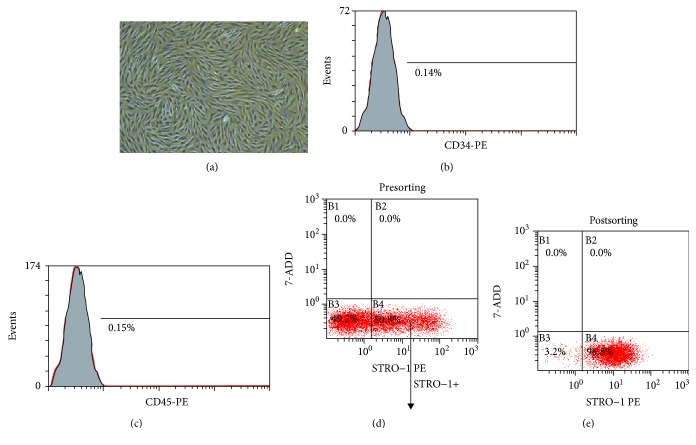
Isolation and identification of PDLSCs. (a) Cells isolated from periodontal ligament tissue show a long and spindle-shaped morphology under phase-contrast microscopy. (b, c) The isolated cells were negative for CD34 and CD45 presented as histogram of flow cytometry analysis. (d) Dot plots represent typical examples of STRO-1 expression and exhibited 45% of STRO-1 positive expression analyzed by flow cytometry. (e) The purity of the sorted population was >96% as revealed by postresorting analysis.

PDLCs isolated from periodontal ligament tissue of healthy individuals possessed long spindle-shaped morphology under a phase-contrast microscopy (Figure 2(a)). Flow cytometry analysis revealed that these isolated cells were negative for HSC markers CD34 (Figure 2(b)) and CD45 (Figure 2(c)). In addition, around 45% of these isolated cells expressed STRO-1 (Figure 2(d)), a well-known MSC surface marker in differentiating to osteoblasts. To obtain homogeneous stem cell population from these isolated cells, FACS was performed to sort STRO-1+ cells that were considered as PDLSCs. The purity of the sorted STRO-1+ population was >96% as revealed by postresorting analysis (Figure 2(e)).

### 3.2. CDR1as Was Downregulated in PDLSCs under an Inflammatory Condition

**Figure 3:**
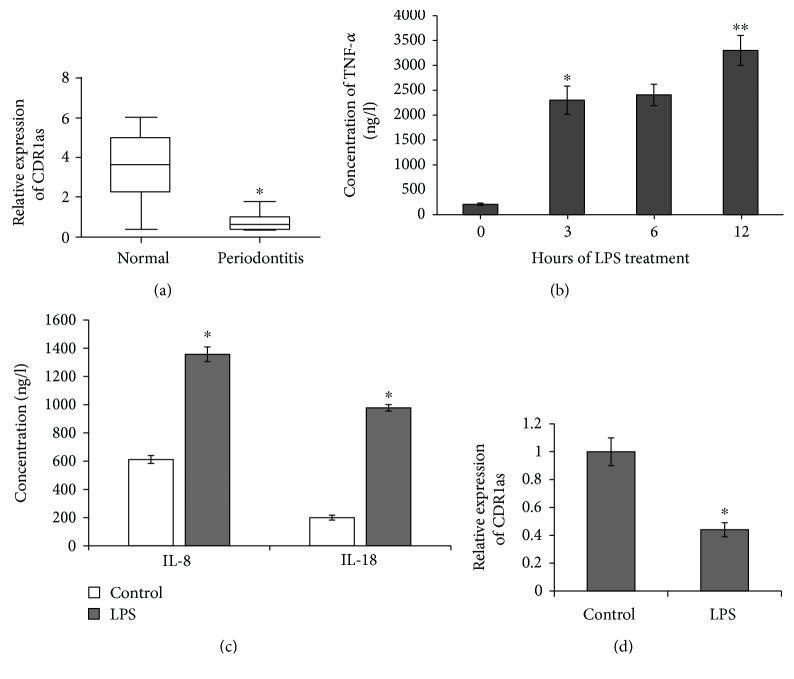
The expression levels of CDR1as in periodontal ligament tissues and PDLSCs. (a) The expression of CDR1as in normal tissues (*n* = 11) and periodontitis tissues (*n* = 10) was determined by RT-qPCR. ^∗^*p* < 0.01 vs. normal. (b) The TNF-*α* protein level secreted in the medium by PDLSCs treated with LPS was measured with an ELISA kit. Untreated PDLSCs (0 h) were used as control. ^∗^*p* < 0.01 vs. control, ^∗∗^*p* < 0.05 vs. 3 h. (c) IL-8 and IL-18 protein levels secreted in the medium by PDLSCs treated with LPS at 10 *μ*g/ml for 3 h were measured with an ELISA kit. Untreated PDLSCs were used as control. ^∗^*p* < 0.01 vs. control. (d) The expression levels of CDR1as in LPS-treated PDLSCs were analyzed by RT-qPCR. Untreated PDLSCs were used as control. ^∗^*p* < 0.01 vs. control.

To explore the potential function of CDR1as during the process of periodontitis, we firstly examined the expression level of CDR1as in human periodontal ligament tissues. We found CDR1as was significantly downregulated in periodontal ligament tissues with periodontitis compared with normal tissues (Figure 3(a)). Moreover, PDLSCs were treated by *P. gingivalis*-derived LPS to mimic an inflammatory condition due to the fact that *P. gingivalis* is one of the main bacteria associated with chronic periodontitis. The expression level of proinflammatory cytokine TNF-*α* was significantly upregulated at 3 h after LPS treatment. No difference was observed between at 3 h and at 6 h after treatment, and there was a highest secretion of TNF-*α* at 12 h (Figure 3(b)). Because LPS induced obvious cell death with 12-hour treatment of LPS, we used the 3-hour treatment of at 10 *μ*g/ml for subsequent experiments. In addition, we found that secretions of another two proinflammatory cytokines IL-8 and IL-18 were significantly upregulated after treatment at 10 *μ*g/ml for 3 h (Figure 3(c)), indicating a successful inflammatory induction of PDLSCs.

Consistent with the expression trend in periodontal ligament tissue, the CDR1as expression level was also significantly lower in PDLSCs under an inflammatory condition than that in normal PDLSCs (Figure 3(d)). These results indicated that decreased expression of CDR1as was critically involved in the process of periodontitis.

### 3.3. CDR1as Mediated LPS-Induced Inhibition of PDLSC Proliferation

**Figure 4:**
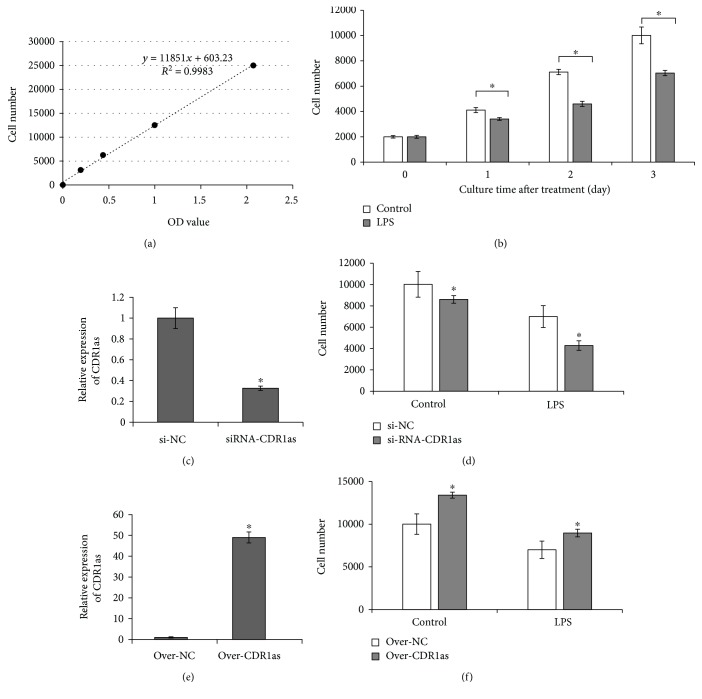
circRNA CDR1as mediated LPS-induced inhibition of PDLSC proliferation. (a) A standard curve of cell proliferation and mathematical formula describing OD value and cell number. Cell proliferation of PDLSCs was assessed by CCK-8 assay as indicated with cell numbers in reference to this standard curve obtained under the same conditions in all subsequent experiments. (b) Cell number of LPS-treated PDLSCs was less than that of untreated cells at each examined day, ^∗^*p* < 0.01. (c) The efficiency of knockdown of CDR1as in PDLSCs was determined by RT-qPCR. ^∗^*p* < 0.01 vs. si-NC. (d) The effects of knockdown of CDR1as on the proliferation of PDLSCs. ^∗^*p* < 0.01 vs. control. (e) The efficiency of overexpression of CDR1as in PDLSCs was determined by RT-qPCR. ^∗^*p* < 0.01 vs. over-NC. (f) The effects of overexpression of CDR1as on the proliferation of PDLSCs, ^∗^*p* < 0.01 vs. control.

The CCK-8 assay was carried out to examine the proliferation of PDLSCs. A standard curve of cell proliferation and mathematical formula describing OD value and cell number were obtained at the same condition (Figure 4(a)). After seeding at the same density in 96-well plates and culturing for 0, 1, 2, and 3 days, the number of PDLSCs with LPS treatment was significantly less than that of the cells without LPS treatment at each indicated day (Figure 4(b)), suggesting that PDLSCs have a lower proliferation capacity under an inflammatory condition induced by LPS than those cells at a normal state. To investigate the functions of CDR1as in PDLSCs during the process of periodontitis, gain- and loss-of-function experiments were performed.

Transfection was conducted to knockdown the expression of CDR1as, and the expression of CDR1as was decreased by approximately 70% in the CDR1as knockdown group compared to the control group as confirmed by RT-qPCR analysis (Figure 4(c)). The proliferation ability was decreased in the CDR1as knockdown group compared to the si-NC group after treatment by LPS (Figure 4(d), right bars). In addition, when cultured with growth medium without LPS treatment, the proliferation ability also decreased in the CDR1as knockdown group compared with the si-NC group (Figure 4(d), left bars).

The pcDNA3.1-circ-mini-CDR1as plasmid was constructed and transfected into PDLSCs. The upregulation of CDR1as in PDLSCs was confirmed by RT-qPCR (Figure 4(e)). CDR1as overexpression significantly promoted PDLSC proliferation in the CDR1as group relative to the circ-control group of over-NC (Figure 4(f), left bars). In addition, CDR1as overexpression partially alleviated LPS-induced proliferative inhibition of PDLSCs (Figure 4(f), right bars).

### 3.4. CDR1as/miR-7 Mediated the LPS-Induced Inhibition of PDLSC Proliferation

Given the fact that CDR1as has been demonstrated to acts as an miR-7 sponge to perform biological functions [12, 13], it is possible that CDR1as may involve LPS-induced proliferative inhibition of PDLSCs via suppressing miR-7 activities.

**Figure 5:**
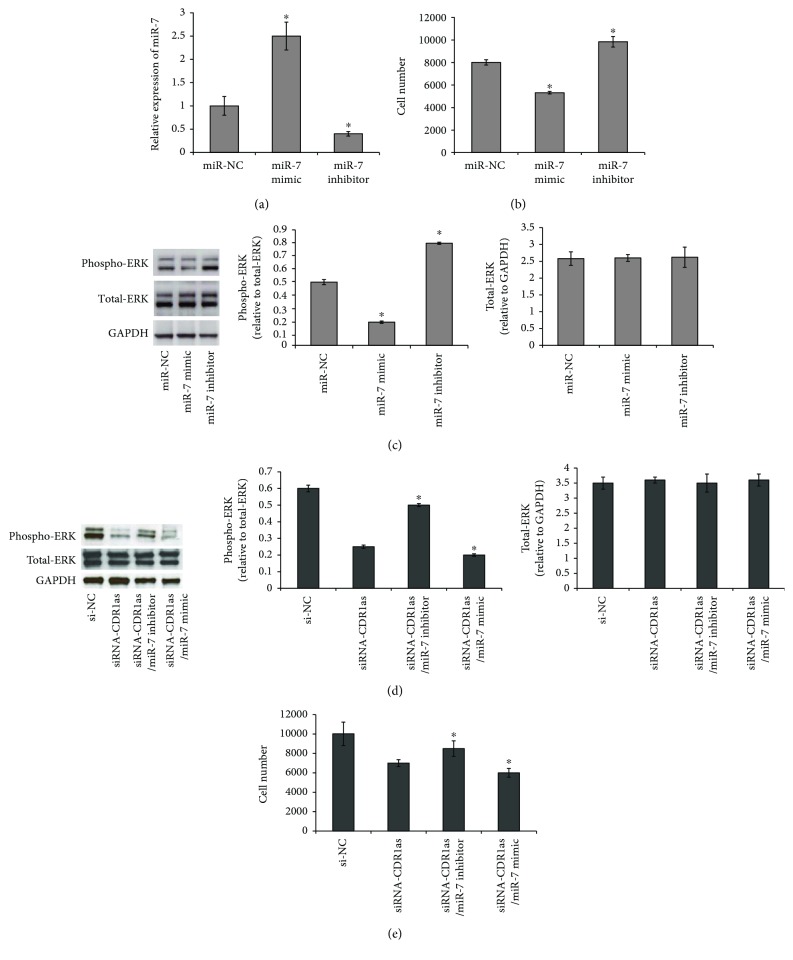
CDR1as/miR-7 regulated LPS-induced inhibition of PDLSC proliferation by targeting ERK. (a) The efficiency of transient transduction of miR-7 mimics and miR-7 inhibitor evaluated by RT-qPCR. ^∗^*p* < 0.01 vs. miR-NC. (b) The effects of miR-1 mimic and inhibitor on the proliferation of PDLSCs. After being transfected with miR-NC, miR-7 mimic, or miR-7 inhibitor, PDLSCs were treated with LPS at 10 ng/*μ*l for 3 h and cultured for another 3 days with an initial seeding density of 2000 cell/well. Cell proliferation was evaluated by CCK-8 kits. ^∗^*p* < 0.01 vs. miR-NC. (c) Western blot analysis of the protein expression of phospho-ERK, total-ERK, and the internal control GAPDH after transfection with miR-7 mimic, miR-7 inhibitor, or miR-NC. ^∗^*p* < 0.01 vs. miR-NC. (d) Western blot analysis of the protein expression of phospho-ERK, total-ERK, and the internal control GAPDH after transfection with siRNA-CDR1as alone or cotransfected with miR-7 inhibit or miR-7 mimic. ^∗^*p* < 0.01 vs. siRNA-CDR1as. (e) The effects of siRNA-CDR1as cotransfected with miR-7 inhibit or miR-7 mimic on the cell proliferation of PDLSCs. ^∗^*p* < 0.05 vs. siRNA-CDR1as.

We firstly investigated whether miR-7 mediates LPS-induced inhibition of PDLSC proliferation. To validate this, we conducted experiments to overexpress or knock down miR-7 by transfections of miR-7 mimic or inhibitor into PDLSCs. Results of RT-qPCR showed that miR-7 was efficiently increased in the miR-7 mimic group and significantly reduced in the miR-7 inhibitory group compared to the miR-NC control group (Figure 5(a)). The proliferation ability was decreased in the miR-7 overexpression group and increased in the miR-7 knockdown group after LPS treatments (Figure 5(b)), suggesting miR-7 did mediate LPS-induced inhibition of PDLSC proliferation.

It has been reported that miR-7 inhibited cell proliferation by targeting the ERK signaling pathway [20, 21]. To validate whether ERK activation acts as a downstream target of miR-7, activation of ERK was investigated by examining the protein expressions of phosphorylated and total ERK in PDLSCs transfected with miR-7 mimic or inhibitor. Results of western blot showed that miR-7 mimic inhibited phosphor-ERK expression, while miR-7 inhibitor enhanced its expression level in PDLSCs compared with those cells in the miR-NC group (Figure 5(c)).

To further demonstrate the link among cell proliferation, CDR1as/miR-7, and ERK, we examined the expressions of phospho-ERK and total-ERK in PDLSCs cotransfected with si-CDR1as and miR-7 mimic or miR-7 inhibitor. We found that cotransfection miR-7 inhibitor with si-CDR1as partially alleviated the si-CDR1as-induced increase of the phospho-ERK expression, and cotransfection miR-7 mimic with si-CDR1as slightly enhanced the inhibitory effect of si-CDR1as on the phospho-ERK expression in PDLSCs (Figure 5(d)). Moreover, cell proliferation ability of PDLSCs was increased in cells cotransfected with si-CDR1as and miR-7 inhibitor, whereas it was decreased in cells cotransfected with si-CDR1as and miR-7 mimic compared to those cells transfected with si-CDR1as alone (Figure 5(e)).

## 4. Discussion

PDLSCs are a unique population of MSCs that possess the capacity to generate cementum- and periodontal ligament-like structures [22]. Therefore, PDLSCs offer potential for the development of novel cell-based therapies to treat the damage caused by trauma or periodontal disease, such as periodontitis [23]. A recent finding suggests that specific circRNAs might function as endogenous RNAs to promote PDLSC periodontal regeneration [6]. In this study, circRNA CDR1as was demonstrated to regulate the proliferation under a LPS-induced inflammatory condition by sponging miR-7 to activate the ERK signal pathway.

PDLSCs have been isolated from human periodontal ligament tissues by using at least two methods, including traditional plastic adherence and immunomagnetic selection based on the expressions of MSC-associated surface markers [22]. Although no antibody demonstrates specificity for MSCs, STRO-1 was the commonly used marker in all the studies related to PDLSC isolation [4]. In this study, we isolated STRO-1+ cells by FACS to get a homogenous population of PDLSCs. All these cells were HSC excluded as indicated by the negative expression of HSC markers CD45 and CD34, and they exhibited MSC-like morphology as described when they were firstly identified [24]. During the process of their periodontal tissue regeneration when inflammation is regarded as the reason for tissue damage, PDLSCs interact with their surrounding inflammatory microenvironment, which could influence their fate, including their stemness, proliferation, migration, differentiation, and immunomodulatory attributes, the possible underlying intracellular mechanisms, and the outcome of any periodontal stem regenerative activity [25]. *P. gingivalis* is one of the main gram-negative bacteria associated with chronic periodontitis [2]. LPS, the major component of the outer membrane of gram-negative bacteria, is a pertinent deleterious factor in the oral microenvironment [26]. Here, we found *P. gingivalis-*derived LPS treatment was able to make PDLSCs under inflammatory condition, as indicated by elevated protein secretion of TNF-*α*, IL-8, and IL-18 by PDLSCs.

CDR1as has been demonstrated to impact biological functions by acting as an miRNA sponge or inhibitor of miR-7 [13], and its functions were clarified to regulate osteoblastic differentiation of PDLSCs via the miR-7/GDF5/SMAD and p38 MAPK signaling pathway [16]. But whether CDR1as has other functions has not been elucidated. In this study, we confirmed that CDR1as was downregulated in periodontal ligament tissues with periodontitis as well as PDLSCs under an inflammatory state induced by *P. gingivalis*-derived LPS compared to their corresponding counterparts, indicating CDR1as may mediate the process of periodontitis and have potential effects on the functions or characteristics of PDLSCs. As we expected, CDR1as mediated LPS-induced inhibition of PDLSC proliferation which was confirmed by gain- and loss-function experiments of CDR1as. CDR1as overexpression alleviated LPS-induced proliferative inhibition of PDLSCs, whereas CDR1as knockdown enhanced this inhibition effect. Given the fact that CDR1as functions as the upstream of miR-7 [16, 27] and miR-7 inhibited cell proliferation by targeting the ERK signaling pathway [20, 21], we further investigated the mechanism in which CDR1as mediated LPS-induced proliferative inhibition of PDLSCs. Mechanically, we found CDR1as functioned as miR-7 sponges to activate the ERK signal pathway to promote PDLSC proliferation. These findings emphasized the functional roles of CDR1as/miR-7 in PDLSCs in an inflammatory state.

## Data Availability

No data were used to support this study.

## Abbreviations

CDR1as: circRNA CDR1as
PDLSCs: Periodontal ligament stem cells
*P. gingivalis*: *Porphyromonas gingivalis*
LPS: Lipopolysaccharide
TNF-*α*: Tumor necrosis factor-alpha
MSC: Mesenchymal stem cell
IL: Interleukin.

## References

[1] Mesa F., Magan-Fernandez A., Castellino G., Chianetta R., Nibali L., Rizzo M. (2019). Periodontitis and mechanisms of cardiometabolic risk: novel insights and future perspectives. *Biochimica et Biophysica Acta - Molecular Basis of Disease*.

[2] Bustamante M., Oomah B. D., Mosi-Roa Y., Rubilar M., Burgos-Diaz C. (2019). Probiotics as an adjunct therapy for the treatment of halitosis, dental caries and periodontitis. *Probiotics and Antimicrobial Proteins*.

[3] Li G., Hu J., Chen H. (2017). Enamel matrix derivative enhances the proliferation and osteogenic differentiation of human periodontal ligament stem cells on the titanium implant surface. *Organogenesis*.

[4] Avinash K., Malaippan S., Dooraiswamy J. N. (2017). Methods of isolation and characterization of stem cells from different regions of oral cavity using markers: a systematic review. *International Journal of Stem Cells*.

[5] Wang T., Kang W., Du L., Ge S. (2017). Rho-kinase inhibitor Y-27632 facilitates the proliferation, migration and pluripotency of human periodontal ligament stem cells. *Journal of Cellular and Molecular Medicine*.

[6] Gu X., Li M., Jin Y., Liu D., Wei F. (2017). Identification and integrated analysis of differentially expressed lncRNAs and circRNAs reveal the potential ceRNA networks during PDLSC osteogenic differentiation. *BMC Genetics*.

[7] Han N., Zhang F., Li G. (2017). Local application of IGFBP5 protein enhanced periodontal tissue regeneration via increasing the migration, cell proliferation and osteo/dentinogenic differentiation of mesenchymal stem cells in an inflammatory niche. *Stem Cell Research & Therapy*.

[8] Zhou Z.-B., Huang G. X., Fu Q. (2019). circRNA.33186 contributes to the pathogenesis of osteoarthritis by sponging miR-127-5p. *Molecular Therapy*.

[9] Yu C.-X., Sun S. (2018). An emerging role for circular RNAs in osteoarthritis. *Yonsei Medical Journal*.

[10] Zhang F., Zhang R., Zhang X. (2018). Comprehensive analysis of circRNA expression pattern and circRNA-miRNA-mRNA network in the pathogenesis of atherosclerosis in rabbits. *Aging*.

[11] Barrett S. P., Salzman J. (2016). Circular RNAs: analysis, expression and potential functions. *Development*.

[12] Sekar S., Cuyugan L., Adkins J., Geiger P., Liang W. S. (2018). Circular RNA expression and regulatory network prediction in posterior cingulate astrocytes in elderly subjects. *BMC Genomics*.

[13] Hansen T. B., Jensen T. I., Clausen B. H. (2013). Natural RNA circles function as efficient microRNA sponges. *Nature*.

[14] Xu H., Guo S., Li W., Yu P. (2015). The circular RNA *Cdr1as*, via miR-7 and its targets, regulates insulin transcription and secretion in islet cells. *Scientific Reports*.

[15] Zhang L., Li Y., Liu W., Li H., Zhu Z. (2018). Analysis of the complex interaction of CDR1as-miRNA-protein and detection of its novel role in melanoma. *Oncology Letters*.

[16] Li X., Zheng Y., Zheng Y. (2018). Circular RNA CDR1as regulates osteoblastic differentiation of periodontal ligament stem cells via the miR-7/GDF5/SMAD and p38 MAPK signaling pathway. *Stem Cell Research & Therapy*.

[17] Yu L., Gong X., Sun L., Zhou Q., Lu B., Zhu L. (2016). The circular RNA Cdr1as act as an oncogene in hepatocellular carcinoma through targeting miR-7 expression. *PLoS One*.

[18] Li P., Yang X., Yuan W. (2018). CircRNA-Cdr1as exerts anti-oncogenic functions in bladder cancer by sponging microRNA-135a. *Cellular Physiology and Biochemistry*.

[19] Tang W., Ji M., He G. (2017). Silencing CDR1as inhibits colorectal cancer progression through regulating microRNA-7. *OncoTargets and Therapy*.

[20] Cao Q., Mao Z. D., Shi Y. J. (2016). MicroRNA-7 inhibits cell proliferation, migration and invasion in human non-small cell lung cancer cells by targeting FAK through ERK/MAPK signaling pathway. *Oncotarget*.

[21] Liu Z., Jiang Z., Huang J. (2014). miR-7 inhibits glioblastoma growth by simultaneously interfering with the PI3K/ATK and Raf/MEK/ERK pathways. *International Journal of Oncology*.

[22] Mrozik K., Gronthos S., Shi S., Bartold P. M. (2017). A method to isolate, purify, and characterize human periodontal ligament stem cells. *Methods in Molecular Biology*.

[23] Zheng W., Wang S., Wang J., Jin F. (2015). Periodontitis promotes the proliferation and suppresses the differentiation potential of human periodontal ligament stem cells. *International Journal of Molecular Medicine*.

[24] Seo B. M., Miura M., Gronthos S. (2004). Investigation of multipotent postnatal stem cells from human periodontal ligament. *The Lancet*.

[25] Fawzy El-Sayed K. M., Elahmady M., Adawi Z. (2018). The periodontal stem/progenitor cell inflammatory-regenerative cross talk: a new perspective. *Journal of Periodontal Research*.

[26] Xing Y., Zhang Y., Jia L., Xu X. (2019). Lipopolysaccharide from *Escherichia coli* stimulates osteogenic differentiation of human periodontal ligament stem cells through Wnt/*β*-catenin-induced TAZ elevation. *Molecular Oral Microbiology*.

[27] Zhang X., Yang D., Wei Y. (2018). Overexpressed CDR1as functions as an oncogene to promote the tumor progression via miR-7 in non-small-cell lung cancer. *OncoTargets and Therapy*.

